# Neonatal pain and *COMT* Val158Met genotype in relation to serotonin transporter (*SLC6A4*) promoter methylation in very preterm children at school age

**DOI:** 10.3389/fnbeh.2014.00409

**Published:** 2014-12-02

**Authors:** Cecil M. Y. Chau, Manon Ranger, Dian Sulistyoningrum, Angela M. Devlin, Tim F. Oberlander, Ruth E. Grunau

**Affiliations:** ^1^Developmental Neurosciences and Child Health, Child and Family Research InstituteVancouver, BC, Canada; ^2^Pediatrics, University of British ColumbiaVancouver, BC, Canada; ^3^Diabetes, Nutrition and Metabolism, Child and Family Research InstituteVancouver, BC, Canada

**Keywords:** preterm, epigenetic, serotonin transporter, catechol-O-methyltransferase, methylation, pain, child, premature

## Abstract

Children born very preterm are exposed to repeated neonatal procedures that induce pain and stress during hospitalization in the neonatal intensive care unit (NICU). The COMT Val158Met genotype is involved with pain sensitivity, and early life stress is implicated in altered expression of methylation of the serotonin transporter. We examined: (1) whether methylation of the serotonin transporter gene (SLC6A4) promoter differs between very preterm children and full-term controls at school age, (2) relationships with child behavior problems, and (3) whether the extent of neonatal pain exposure interacts with the COMT Val158Met genotype to predict SLC6A4 methylation at 7 years in the very preterm children. We examined the associations between the COMT genotypes, neonatal pain exposure (adjusted for neonatal clinical confounders), SLC6A4 methylation and behavior problems. Very preterm children had significantly higher methylation at 7/10 CpG sites in the SLC6A4 promoter compared to full-term controls at 7 years. Neonatal pain (adjusted for clinical confounders) was significantly associated with total child behavior problems on the Child Behavior Checklist (CBCL) questionnaire (adjusted for concurrent stressors and 5HTTLPR genotype) (*p* = 0.035). CBCL Total Problems was significantly associated with greater SLC6A4 methylation in very preterm children (*p* = 0.01). Neonatal pain (adjusted for clinical confounders) and COMT Met/Met genotype were associated with SLC6A4 promoter methylation in very preterm children at 7 years (*p* = 0.001). These findings provide evidence that both genetic predisposition and early environment need to be considered in understanding susceptibility for developing behavioral problems in this vulnerable population.

## Introduction

Early life adversity and stress exposure are associated with alterations in DNA methylation and changes in gene expression (McGowan et al., 2009; Meaney, 2010; Bagot et al., 2012) and importantly, such changes may be already taking shape long before birth (Oberlander et al., 2008). DNA methylation is a critical epigenetic mechanism regulating gene expression and has been suggested as a possible molecular mechanism explaining the diversity in behavioral outcomes that follow exposure to early life stressful events (Caspi et al., 2003; Rutter et al., 2006; McGowan et al., 2009). DNA methylation offers a promising direction that enables us to understand how exposure to adverse/stressful early life events may lead to enduring changes in neuronal function (Karsten and Baram, 2013).

Infants born very preterm [24–32 weeks gestational age (GA)] spend weeks to months in the neonatal intensive care unit (NICU) during a delicate and critical phase of very rapid brain development, and programming of stress systems (Grunau et al., 2004; Tu et al., 2007). In the NICU, these infants require numerous invasive medical procedures to diagnose and treat life-threatening conditions. This environment contrasts greatly from the calm and protective maternal intrauterine environment, thus exposing these fragile neonates to repeated highly stressful and potentially painful experiences that are developmentally unexpected. Since stress and pain cannot be distinguished in immature neonates, we have used the term “pain-related stress.” Despite major improvement in the care of very preterm infants, rates of neurodevelopmental problems in children born very preterm do not appear to have improved, affecting about 50% of survivors (Gray et al., 2004; Aarnoudse-Moens et al., 2009). There are concerns regarding long-term consequences of pain-related stress in very preterm neonates. Recent work from our group found that procedural pain/stress exposure (controlling for clinical factors related to prematurity, such as gestational age, postnatal infection, cumulative morphine dose, days of mechanical ventilation, illness severity on day 1, and number of surgeries) was associated with altered gray and white matter maturation (Brummelte et al., 2012; Ranger et al., 2013; Vinall et al., 2013a), corticospinal tract development (Zwicker et al., 2013a,b), poorer motor (Zwicker et al., 2013a), cognitive (Grunau et al., 2009; Doesburg et al., 2013), and behavioral functioning in very preterm children (Vinall et al., 2013b; Ranger et al., 2014). Moreover, greater cumulative exposure to neonatal pain/stress has been associated with altered developmental trajectory of stress hormone (cortisol) expression [i.e., programming of the hypothalamic-pituitary-adrenal axis (HPA)] long after NICU discharge (Grunau et al., 2007, 2013; Miller et al., 2011). Examining the role of gene and environment interactions is important to understand mechanisms and the etiology of how early pain/stress contributes to altered neurodevelopment in very preterm children.

We have taken a hypothesis-based targeted gene approach. Two key stress and mood regulatory neurotransmitters and related regulatory genes have been identified: serotonin and dopamine. Serotonin is a widely distributed neurotransmitter that appears early in gestation that plays a key role in early brain development and later emotion, attention, cognition, and learning during childhood (Lucki, 1998; Lesch, 2007; Weikum et al., 2013). Central to the regulation of intrasynaptic serotonin (5-hydroxytryptamine, 5HT) concentrations is the presynaptic membrane-bound serotonin transporter protein (5HTT, encoded by *SLC6A4*). Variants in the serotonin transporter promoter region (*5HTTLPR*), are associated with altered gene expression and 5HTT concentrations, and therefore have a critical role in determining intrasynaptic serotonin signaling (Hanley and Oberlander, 2012). The short (S) allele of *5HTTLPR* results in ~50% reduction in serotonin reuptake compared to the long (L) allele (Heils et al., 1996). S allele carriers may have greater vulnerability for emotional disorders (Homberg and Lesch, 2011), particularly in the context of early life stress (Caspi et al., 2003) (recent reviews Serretti et al., 2007; Kuzelova et al., 2010). Manipulations of 5HT signaling, either via pharmacologic 5HTT blockade or *SLC6A4* knock-out in mouse models, that increase serotonin concentrations during developmentally sensitive periods, are associated with lasting behavioral, neurophysiological, and neuroanatomical changes in animal models (Homberg et al., 2010; Olivier et al., 2011; Simpson et al., 2011) and increased risk for anxiety/depression symptoms in humans (Oberlander et al., 2010; Weikum et al., 2013). It is well established that behavioral problems such as attention deficit problems, internalizing (depressive/anxiety symptoms), and to some extent externalizing (aggressive, hyperactivity symptoms) behaviors are prevalent in children born very preterm (Bhutta et al., 2002; Gray et al., 2004; Aarnoudse-Moens et al., 2009). Recently, methylation of the *SLC6A4* promoter was shown to be associated with early childhood adversity in adults with major depression (Kang et al., 2013). Therefore, examining the impact of early life stress on changes in the methylation of the *SLC6A4* promoter in very preterm children is likely to add to our understanding of how early changes in serotonin signaling might be associated with altered neurodevelopmental outcomes differences previously reported in this population compared to term born counterparts.

Catechol-*O*-transferase (*COMT)* gene encodes for a key enzyme in the metabolism of dopamine (DA), norepinephrine (NE), and epinephrine (EPI) (Lotta et al., 1995). The Met/Met genotype of the *COMT* Val158Met variant is associated with more than a three- to four-fold decrease in COMT enzyme activity and DA catabolism, which leads to an increase in DA availability and reduced myelination (Lotta et al., 1995; Chen et al., 2004). This is especially significant in the prefrontal cortex (PFC) where COMT activity is the primary determinant of synaptic DA concentrations (Garris and Wightman, 1994; Karoum et al., 1994). *COMT* variants have been associated with cognitive functions (Blasi et al., 2005; Dumontheil et al., 2011); and mood disorders (Enoch et al., 2003; Olsson et al., 2007; Aberg et al., 2011). For example, individuals with the Met allele are at increased risk for anxiety (Enoch et al., 2003; Olsson et al., 2007).

Moreover, Zubieta and colleagues reported effects of the *COMT* Val158Met genotype on μ-opioid system activation in young adults, where individuals with the Met/Met genotype had greater pain sensitivity and negative emotions to pain compared to individuals with the Met/Val and Val/Val genotypes (Zubieta et al., 2003). Further, the Val allele has been associated with increased risk of schizophrenia in both European and Asian populations (Fan et al., 2005), and decreased cognitive stability (Bialecka et al., 2008). COMT and 5HTT have important functional roles in regulating neurotransmission with mood and behavior brain-related pathways, and given that both have functional genetic variants, researchers have begun to investigate their potential interaction in various pathologies (Olsson et al., 2007; Radua et al., 2014).

Since both dopaminergic and serotonergic systems play a critical role in mood regulation, there has been a considerable amount of interest in their potential interactions. Drugs enhancing dopaminergic function such as buproprion are also effective antidepressants (Ascher et al., 1995). In rodents, exposure of the striatum and nucleus accumbens to serotonin increased dopamine release (review by Sasaki-Adams and Kelley, 2001). Dopamine and serotonin systems also showed a significant degree of convergence and plasticity in the rat medial prefrontal cortex (Benes et al., 2000).

To further understand how early life stress exposure may contribute to altered behavior in children born very preterm, this study was undertaken to determine if methylation of *SLC6A4* promoter differed between very preterm children and full-term controls at age 7 years. We hypothesized that methylation at school age would be altered in those born very preterm due to the stressful neonatal environment. We further explored whether child behavioral problems, concurrent child stress level, and parental factors were associated with differences in methylation. To investigate the effects of neonatal stress on child behavioral problems and *SLC6A4* methylation, in very preterm group only, we examined whether neonatal procedural pain exposure (adjusted for clinical confounders) and/or concurrent stressors are associated with child behavioral problems at age 7 years. Finally, in very preterm group only, we examined the effect of neonatal procedural pain exposure (adjusted for clinical confounders) and *COMT* Val158Met genotype on *SLC6A4* methylation at 7 years. Given that *COMT* Val158Met genotype has been reported to be associated with cognitive functions and pain sensitivity, we hypothesized that the extent of early pain exposure would interact with the *COMT* Val158Met genotype to affect *SLC6A4* methylation.

## Materials and methods

### Participants

Participants were part of a larger longitudinal study of long-term effects of neonatal pain-related stress on brain, stress and neurodevelopment of children born very preterm (24–32 weeks gestation) (e.g., Grunau et al., 2007, 2009), who were admitted to the level III NICU at Children's and Women's Health Centre of British Columbia between 2000 and 2004. A total of 111 school age children, 61 born very preterm and 50 born full-terms were included in the current study [56 boys/55 girls; mean age 7.78 y (±0.65) range 6.3–10.8 years]. Parents completed questionnaires while children were undergoing psychometric testing.

All of the children had an IQ score above 70 on the Wechsler Intelligence Scale for Children—4th Ed (Wechsler, 2003). None had major cognitive, sensory, motor impairments, or was diagnosed with autism. We included in our sample three very preterm children with intraventricular hemorrhage (IVH) grade 1 or 2, and two grade 3 or 4, on neonatal ultrasound. This study was approved by the Clinical Research Ethics Board of the University of British Columbia and the Research Ethics Board of the B.C. Children's and Women's Hospitals, and conforms to the conventions set out in the Declaration of Helsinki. Written informed consent was obtained from parents and assent from children.

### Neonatal data

Medical and nursing chart review of children born very preterm was carried out by an experienced neonatal research nurse. The data collection included but was not limited to, GA, birth weight, illness severity on day 1 [Score for Neonatal Acute Physiology (SNAP)- II (Richardson et al., 2001)], days of mechanical ventilation, presence of culture proven infection, number of surgeries, and cumulative dose of morphine (**Table 2**). We quantified neonatal pain-related stress as the number of skin-breaking procedures (e.g., heel lance, peripheral intravenous or central line insertion, chest-tube insertion, and nasogastric tube insertion) during the stay in the NICU, as previously reported (e.g., Grunau et al., 2009; Brummelte et al., 2012). Each attempt at a procedure was counted as one skin-break; all nursing staff in our NICU have been trained to precisely record each attempt. Cumulative morphine exposure was calculated as the average daily dose (i.e., intravenous dose plus intravenous-equivalent oral dose) adjusted for daily body weight, multiplied by the number of days morphine was administered.

### Measures/assessments

Children completed the Wechsler Intelligence Scale for Children—Fourth Edition (WISC-IV) (Wechsler, 2003). A parent (110 mothers, 1 father) completed the following questionnaires:

- Child Behavior Checklist (CBCL) for children ages 6–18 years (Achenbach and Rescorla, 2000), the most widely used questionnaire for identifying problem behaviors in children. Ratings are on a 3-point Likert scale [ranging from 0 (not true) to 2 (very true or often true)] on 113 items regarding child behavior in the past 6 months. The checklist is comprised of eight syndrome scales (Anxious/Depressed, Withdrawn/Depressed, Social Problems, Somatic Complaints, Thought Problems, Attention Problems, Aggressive Behavior, and Rule-Breaking Behavior) and two higher order-factors of Internalizing and Externalizing Problems. Raw scores were converted to age-standardized scores (T scores with mean = 50 and *SD* = 10) based on the normative samples of children for age range separately by sex (Achenbach and Rescorla, 2000). The Total Problems T-score was used as an indicator of overall emotional and behavioral problems of the child, and yields an alpha coefficient of 0.97 (Achenbach and Rescorla, 2000).
- The Beck Depression Inventory—2nd Editions (BDI-II) (Beck et al., 1961) a 21-item self-report multiple-choice questionnaire widely used to assess the presence and severity of symptoms of depression, was used to evaluate parent depression. The scale yields a single score that ranges from 0 to 63, with higher scores indicating higher level of depression; reliability alpha coefficient 0.92 (Gatewood-Colwell et al., 1989).
- The State-Trait Anxiety Inventory (Spielberger et al., 1983) comprised of two separate 20-statement self-report scales: the State-Anxiety scale to evaluate how respondents feel “right now, at this moment” and the Trait-Anxiety scale how respondents generally feel. Only the Trait Anxiety Scale was used in the present study as an indicator of parent general anxiety symptoms. Scores range from 20 to 80; reliability alpha coefficient 0.86 (Spielberger et al., 1983).

### Hair cortisol

Cortisol was assayed from hair as an integrated measure of stress level (HPA activity) in the previous 2 months following our published protocol (Grunau et al., 2013; Vaghri et al., 2013). Hair samples were collected from the vertex posterior of the head, as this area has been shown to have the lowest coefficient of variation in hair cortisol concentrations (Sauve et al., 2007). A cluster of hair strands (~2–3 mm diameter) was cut at the base of the hair shaft from five small spots. The hair samples were secured on a cardboard card with tape, labeled, and stored in individual sealed plastic bags at room temperature until analysis. The most proximal 2 cm from the scalp of each hair sample was utilized for the assay. Ten to 15 mg from each hair sample was placed in scintillation vials. To remove external contaminants, hair samples were washed twice by immersion in 3 mL of isopropanol and incubating at room temperature in a centrifuge at 100 rpm for 3 min. After decanting the isopropanol, samples were allowed to dry for at least 12 h. After drying, 1 mL of methanol was added to each scintillation vial. Hair was then finely minced with surgical scissors. The vials were sealed with paraffin film and incubated for 16 h at 50°C in a centrifuge at 100 rpm. Following methanol extraction each cortisol-containing methanol solution was transferred into 5 mL test tubes and evaporated on a test tube hot plate under a steady stream of nitrogen gas. The residue was then reconstituted with 250 μL of phosphate buffered saline. These reconstituted samples were analyzed using the salivary enzyme linked immunoassay kit (Alpco Diagnostics, Salem, NH, USA). The intra-assay and inter-day coefficients of variation were 8.9 and 5.1%, respectively. The kit reported a sensitivity of 1.0 ng/mL.

### Genotyping

Genomic DNA was extracted from saliva using Oragene OG-500 and prepIT-L2P collection kit (DNA Genotek/OraSure Technologies Inc., Bethlehem, Pennsylvania). The *COMT* Val158Met (rs4680) variant was genotyped using TaqMan SNP Genotyping Assay reagents and an Applied Biosystems 7300 Real Time PCR System (Applied Biosystems, Carlsbad, California). Call rate was 100%. The Hardy-Weinberg equilibrium of allelic distribution of the cohort was examined and compared to the population distribution by chi-square test.

The L and S alleles of the *5HTTLPR* serotonin transporter variant was genotyped by polymerase chain reaction (PCR) with the following primers stpr5, 5′-GGCGTTGCCGCTCTGAATGC and stpr3, 5′-GAGGGACTGAGCTGGACAACCAC to generate a 484-bp (S short allele) or a 528-bp (L long allele) product, distinguished by agarose gel electrophoresis.

### Epigenetic analysis

Using the DNA from saliva, the methylation status of a CpG-rich region in the *SLC6A4* promoter was quantified by bisulfite Pyrosequencing (Dupont et al., 2004) as previously described (Devlin et al., 2010). This region of the *SLC6A4* promoter has been reported to be differentially methylated and associated with changes in *SLC6A4* mRNA expression (Philibert et al., 2007, 2008; Devlin et al., 2010). Briefly, we have analyzed a region of the *SLC6A4* promoter between −479 and −350, relative to the transcriptional start site, which contains 10 CpG sites and is adjacent to exon 1a (Devlin et al., 2010). Genomic DNA was bisulfite-treated using the EZ DNA Methylation Gold Kit (Zymo Research) following the manufacturer's instructions and stored at −20°C until further analyzed. A 130 bp fragment of the *SLC6A4* promoter was amplified by PCR from bisulfite-treated DNA using HotStar Taq DNA Polymerase (Qiagen) and the following primers for SLC6A4: PMHSERTF, 5′-GTATTGTTAGGTTTTAGGAAGAAAGAGAGA-3′ and PMHSERTR, 5′-AAAAATCCTAACTTTCCTACTCTTTAACTT-3′ (IDT Inc, Coralville, IA), with the reverse primer containing a biotin at the 5′ end. PCR products were purified and sequenced using a PyroMark MD System (Biotage, Foxboro, MA) following the manufacturer's suggested protocol and the following sequencing primer PMHSERTS, 5′-AAACTACACAAAAAAACAAAT-3′ (IDT). The percent methylation at each CpG site was quantified using the Pyro Q-CpG software, version 1.0.9 (Biotage).

### Statistical analysis

*T*-tests were used to compare child characteristics between very preterm and full-term groups. The number of skin-breaking procedures was log-transformed and shown to be normally distributed by the Komogorov-Smirnov Normality Test. Percent Methylation of the 10 *SLC6A4* CpG sites were arcsine transformed to address the issue of heteroscedasticity (Lin et al., 2008; Laird, 2010). Principal component analysis (PCA) was used to reduce the data in contrast to using the mean methylation of selected CpG sites because of significant variances. This has been used by others to examine SLC6A4 promoter methylation; the first PCA factor had the largest possible variance and was extracted to represent the overall SLC6A4 promoter methylation of the 10 CpG sites in the dataset (Beach et al., 2010). To examine the relationship between *COMT* Val158Met genotypes and cumulative neonatal skin-breaking procedures (adjusted for clinical confounders), generalized linear modeling (GZLM) was used. GLZM provides an extension of general linear models and relaxes the requirement of equality or constancy of variances that is required in traditional linear models. Statistical analyses were performed using the Statistical Package for Social Sciences (SPSS) version 22 (IBM, Somers, NY); *p* < 0.05 were considered statistically significant.

## Results

### Participant characteristics

Child and parent characteristics of 111 school-age children (61 born very preterm; 50 born full-term) are summarized in Table 1A. Parents of very preterm children had significantly higher self-reported depression (on the Beck Depression Inventory), compared to parents of full-term children (*p* = 0.02). Children born very preterm (in this sample that excluded children with low IQ or major brain injury and sensory-motor impairments) showed a trend toward higher Total Problems on the Child Behavior Check List completed by parents, compared to full-terms (*p* = 0.055). Neonatal characteristics of the very preterm children are shown in Table 1B. Genotype frequencies of *COMT* Val158Met and the *5HTTLPR* variant are shown in Table 2. Due to the low frequencies of the S allele for *5HTTLPR*, subjects with homozygous S alleles (S/S) and the L/S genotype were grouped together in all analyses. There were no significant differences in genotype frequency between very preterm and full-term children, and the distribution of *5HTTLPR* and *COMT* Val158Met genotypes had no significant discrepancy from the Hardy-Weinberg equilibrium (*p* = 0.21 and *p* = 0.36 respectively).

**Table 1A:** **Child and parent characteristics**.

	**Very preterm (*N* = **61**) mean (*SD*)**	**Full-term (*N* = **50**) mean (*SD*)**	***p*-Value**
Child age (years)	7.7 (0.3)	7.9 (0.9)	0.090
Child hair cortisol (ng/ml)	2.33 (0.35)	2.38 (0.33)	0.468
Child CBCL total problems T score	51.3 (10.4)	47.5 (10.0)	**0.055**
Child CBCL internalizing behavior T score	51.3 (10.3)	50.3 (10.7)	0.616
Child CBCL externalizing behavior T score	48.7 (9.6)	47.9 (10.3)	0.694
Parent BDI-II	6.9 (8.0)	4.0 (4.6)	**0.020**
Parent STAI trait anxiety	35.4 (9.7)	34.2 (8.3)	0.471

*SD, standard deviation; CBCL, Child Behavior Check List; BDI-II, Beck Depression Inventory 2nd edition; STAI, State Trait Anxiety Inventory*.

**Table 1B:** **Neonatal characteristics of children born very preterm**.

	**Mean (*SD*)**	**Range**
Male (number, %)	36 (59)	
Gestational age at birth (weeks)	28.2 (2.1)	25.0–32.9
Birth weight (g)	1134 (390)	520–2265
Skin-breaks	142 (89)	30–446
Cumulative morphine dose (mg)	2.27 (4.8)	0–22.9
Days of mechanical ventilation	16 (17)	1–58
Illness severity day I (SNAP-II)	15.7 (11.6)	0–45
Number of surgeries	0.5 (0.8)	0–3
Postnatal infection (number, %)	27 (44)	

*SD, standard deviation; SNAP-II, score for neonatal acute physiology (severity of illness index)*.

*Skin-breaks (i.e., neonatal pain-related stress) is equal to the number of skin-breaking procedures from birth to term equivalent age or hospital discharge; cumulative morphine dose is equal to daily morphine exposure adjusted for daily weight; postnatal infection is counted as the presence of a culture proven infection*.

**Table 2:** **Allelic distribution of *COMT* and *SERT* genotypes**.

	**Genotype**	**Preterm**	**Full-term**	**Chi-square *p*-value^a^**	**Hardy-Weinberg equilibrium *p*-value^b^**
SERT 5*HTTLPR*	LL	19	10	0.17	0.21
	LS +SS	41	40		
*COMT* rs4680 (C_25746809_50)	GG Val/Val	20	13	0.78	0.36
	AG Val/Met	30	26		
	AA Met/Met	10	9		

a *p-Values from Pearson Chi-Square tests between preterm and full-term children*.

b *p-Values from Hardy-Weinberg Equilibrium between allelic distributions*.

*L, Long allelic variant of SLC6A4 promotor; S, Short allelic; SERT, serotonin promoter gene; COMT, Catechol-O-methyltransferase*.

### SLC6A4 methylation in very preterm and full-term children

*T*-tests with correction for multiple comparisons were used to compare the transformed methylation in the 10 *SLC6A4* CpG sites (CpG 1–10) between the very preterm and full-term groups. Seven out of the 10 *SLC6A4* CpG sites had significantly higher methylation (CpG 1, 3–5, 8–10) and CpG 2 had lower methylation in very preterm children compared to full-term children (Table 3A). CpG 1–5 remained significant after correction for multiple comparisons. Overall, at age 7 years, children born very preterm had higher *SLC6A4* methylation compared to children born full-term.

**Table 3A:** ***SLC6A4* percent methylation in preterm and full-term children**.

***SLC6A4 CpG sites***	Preterm (*N* = **61**) mean (SD^*^)	**Full-term (*N* = **50**) mean (*SD*)**	*p*-Value^**^
CpG 1	3.79 (0.92)	3.15 (0.72)	**<0.0001^†^**
CpG 2	1.60 (0.66)	2.00 (0.44)	**<0.0001^†^**
CpG 3	4.96 (1.06)	4.17 (0.74)	**<0.0001^†^**
CpG 4	1.79 (0.60)	1.32 (0.52)	**0.001^†^**
CpG 5	4.06 (0.87)	2.69 (0.69)	**<0.0001^†^**
CpG 6	1.49 (0.60)	1.62 (0.43)	0.142
CpG 7	2.87 (0.75)	2.93 (0.70)	0.777
CpG 8	3.48 (0.88)	3.09 (0.65)	**0.009**
CpG 9	3.52 (0.81)	3.11 (0.73)	**0.008**
CpG 10	6.08 (1.43)	5.42 (1.23)	**0.017**

* *SD, standard deviation of % methylation*.

** *p-Values of t-tests of the arcsine transformed SLC6A4 methylation [sin^−1^ (% methylation/100)^*1/2*^] between the preterm and full-term groups*.

† *Significant after controlled for multiple comparisons by Bonferroni correction*.

### Principal component analysis of SLC6A4 methylation

Principal component analysis (PCA) was used for data reduction. Three principal components had an eigenvalue > 1, and together they accounted for 57% of the total variance in *SLC6A4* methylation. Principal Component 1 (PC1) (eigenvalue = 3.2) explained 32% of variance, while PC2 (eigenvalue = 1.4) and PC3 (eigenvalue = 1.1) explained 15 and 10% respectively. Loadings of the CpG sites on the three components are shown in Table 3B. As PC1 was the predominant factor on the PCA, it was used to represent the most significant aspect of methylation in *SLC6A4* CpG 1–10 in the present study.

**Table 3B:** **PCA component loadings of 10 CpG sites**.

***SLC6A4* CpG sites**	**Component 1**	**Component 2**	**Component 3**
CpG 1	0.653	−0.120	0.018
CpG 2	−0.107	0.746	0.247
CpG 3	0.591	−0.029	0.404
CpG 4	0.541	−0.466	0.217
CpG 5	0.763	−0.408	0.031
CpG 6	0.031	0.225	0.801
CpG 7	0.527	0.453	−0.185
CpG 8	0.624	0.142	−0.018
CpG 9	0.748	0.269	−0.174
CpG 10	0.546	0.372	−0.278

*CA, principal component analysis*.

*Component 1 (eigenvalue = 3.2) explained 32% of variance*.

*Component 2 (eigenvalue = 1.4) explained 14% of variance*.

*Component 3 (eigenvalue = 1.1) explained 11% of variance*.

To investigate the effects of neonatal pain-related stress on child behavioral problems and *SLC6A4* methylation, we examined the relationships between: (1) *SLC6A4* methylation and child behavior problems; (2) number of neonatal skin-breaking procedures (adjusted for other neonatal factors) and child behavior problems; and (3) number of skin-breaking procedures (adjusted for other neonatal factors) and *SLC6A4* methylation in very preterm children.

### SLC6A4 methylation and child behavior problems

To investigate factors contributing to the altered *SLC6A4* methylation in very preterm children, GZLM analyses were then performed separately in very preterm and full-term children to determine the relationship with *SLC6A4* methylation. Separate analyses were conducted for very preterm and full-term children since only the very preterm children were exposed to the early adversity of procedural pain, therefore we expected different relationships between the predictor variables and *SLC6A4* methylation for each group. To build the group factor into the GZLM would require adding too many interaction terms. The CBCL Total Problems T score, concurrent parental depressive symptoms (BDI II) and trait anxiety (STAI), hair cortisol concentration as an index of cumulative stress level and perceived stress of the child, and *5HTTLPR* genotype (LL, LS, and SS) were entered simultaneously as covariates to determine the effect on *SLC6A4* methylation (Component 1 in PCA). *5HTTLPR* genotype was included as a covariate because it may contribute to *SLC6A4* methylation differences (Beach et al., 2014). In very preterm children, higher CBCL Total Problems score was significantly related to greater *SLC6A4* methylation (*r*^2^ = 0.13; *p* = 0.01) after adjusting for the other concurrent factors. For the full-term children, since the Omnibus test was not statistically significant, there was no further interpretation. Results of the GZLM are summarized in Tables 4A,B.

**Table 4A:** **Generalized linear modeling analysis to predict *SLC6A4* methylation in very preterm children**.

**Parameter**	**B^a^**	**Std. error**	**95% confidence interval**		***p*-Value**
			**Lower**	**Upper**	
CBCL total problems T-score	0.042	0.0161	0.010	0.074	**0.010**
Child hair cortisol level	0.024	0.4018	−0.764	0.811	0.953
Parent BDI-II	−0.037	0.0293	−0.094	0.020	0.207
Parent STAI trait anxiety score	0.023	0.0216	−0.020	0.065	0.295
Child *5HTTLPR* LL	0.577	0.3048	−0.020	1.175	0.058

*Omibus test: p = 0.035*.

*GZLM results showing CBCL Total Problem T-score (adjusted for concurrent stressors, hair cortisol, and 5HTTLPR genotype) associated with SLC6A4 methylation in children born very preterm*.

*Model: SLC6A4 methylation = CBCL Total Problem T-score + Child hair cortisol + BDI-II + STAI anxiety + 5HTTLPR genotypes*.

a *Unstandardized regression coefficient*.

*CBCL, Child Behavior Check List; BDI-II, Beck Depression Inventory 2nd edition; STAI, State Trait Anxiety Inventory; L, Long allelic variant of SLC6A4 promotor; S, Short allelic*.

**Table 4B:** **Generalized linear modeling analysis to predict *SLC6A4* methylation in full-term children**.

**Parameter**	**B^a^**	**Std. error**	**95% confidence interval**		***p*-Value**
			**Lower**	**Upper**	
CBCL total problems T-score	0.040	0.0190	0.003	0.077	0.036
Child hair cortisol level	0.316	0.4928	−0.649	1.282	0.521
Parent BDI-II	0.027	0.0444	−0.060	0.113	0.550
Parent STAI trait anxiety score	−0.023	0.0272	−0.076	0.031	0.406
Child *5HTTLPR* LL	0.377	0.3951	−0.398	1.151	0.340

*Omibus test: p = 0.314*.

*GZLM results showing CBCL Total Problems T-score (adjusted for concurrent stressors, hair cortisol, and 5HTTLPR genotype) associated with SLC6A4 methylation in children born very preterm*.

*Model: SLC6A4 methylation = CBCL Total Problems T-score + Child hair cortisol + BDI-II + STAI anxiety + 5HTTLPR genotypes*.

a *Unstandardized regression coefficient*.

*CBCL, Child Behavior Check List; BDI-II, Beck Depression Inventory 2nd edition; STAI, State Trait Anxiety Inventory; L, Long allelic variant of SLC6A4 promotor; S, Short allelic*.

*COMT* Val158Met was included in a separate model since this genotype may be associated with emotion and behavior regulation in children (Albaugh et al., 2010; Sheikh et al., 2013); the *COMT* genotype had no significant relationship with methylation (*p* = 0.518). Also, alternative models were built separately to examine whether CBCL Externalizing and Enternalizing T scores were associated with *SLC6A4* methylation. Before adjusting for multiple comparisons Externalizing (*p* = 0.035) and Internalizing (*p* = 0.077) were marginally significant, however neither was significant after adjusting for multiple comparisons of Externalizing and Internalizing scores.

### Neonatal pain and child behavior problems

To further advance our understanding of neonatal pain in relation to child behavior problems at 7 years, GZLM was used to investigate the association with CBCL Total Problems T score. Predictors were neonatal pain (cumulative skin breaking procedures from birth to term equivalent age) adjusted for clinical confounders related to prematurity (GA at birth, number of days on mechanical ventilation, illness severity on day 1 [SNAP-II], number of surgeries, presence of culture proven infection, cumulative dose of morphine), and concurrent parental depressive symptoms (BDI II) and trait anxiety (STAI), and child stress level (hair cortisol) as covariates. Greater neonatal pain (after adjustment for all covariates) was associated with higher Total Problems score on the Child Behavior Checklist (CBCL) (*p* = 0.035, *r*^2^ = 0.39). *5HTTLPR* and *COMT* genotypes were then added in subsequent models and were not significant (*p* = 0.866). GZLM results are summarized in Table 5.

**Table 5:** **Generalized linear modeling analysis of CBCL Total Problems T scores predicted by neonatal pain-related stress**.

**Parameter**	**B^a^**	**Std. error**	**95% confidence interval**		***p*-Value**
			**Lower**	**Upper**	
Skin-breaks	17.99	8.54	1.25	34.73	**0.035**
Neonatal infection	3.80	2.89	−1.86	9.47	0.188
Cumulative morphine dose	8.88	6.41	−3.68	21.44	0.166
Days of ventilation	−0.04	0.16	−0.35	0.27	0.779
SNAP-II day 1	0.14	0.12	−0.11	0.38	0.279
Number of surgery	−2.24	1.93	−6.03	1.55	0.246
Gestational age (GA)	2.93	0.88	1.21	4.65	**0.001**
Child hair cortisol level	2.18	3.36	−4.40	8.76	0.516
Parent BDI-II	0.56	0.25	0.07	1.06	**0.025**
Parent STAI trait anxiety score	−0.01	0.18	−0.37	0.35	0.959

*GZLM results showing CBCL Total Problems T scores was predicted by neonatal pain-related stress*.

*Model: CBCL Total Problems T scores = skin-breaks + Infection + morphine exposure + days of ventilation + SNAP-II + surgery + GA + Child hair cortisol + BDI-II + STAI anxiety*.

a *Unstandardized regression coefficient*.

*SNAP-II, score for neonatal acute physiology (severity of illness index)*.

*Skin-breaks (i.e., neonatal pain-related stress) is equal to the number of skin-breaking procedures from birth to term equivalent age or hospital discharge; cumulative morphine dose is equal to daily morphine exposure adjusted for daily weight; postnatal infection is counted as the presence of a culture proven infection*.

### Effect of neonatal pain and COMT Val158Met genotypes on SLC6A4 methylation in very preterm children

**Figure 1:**
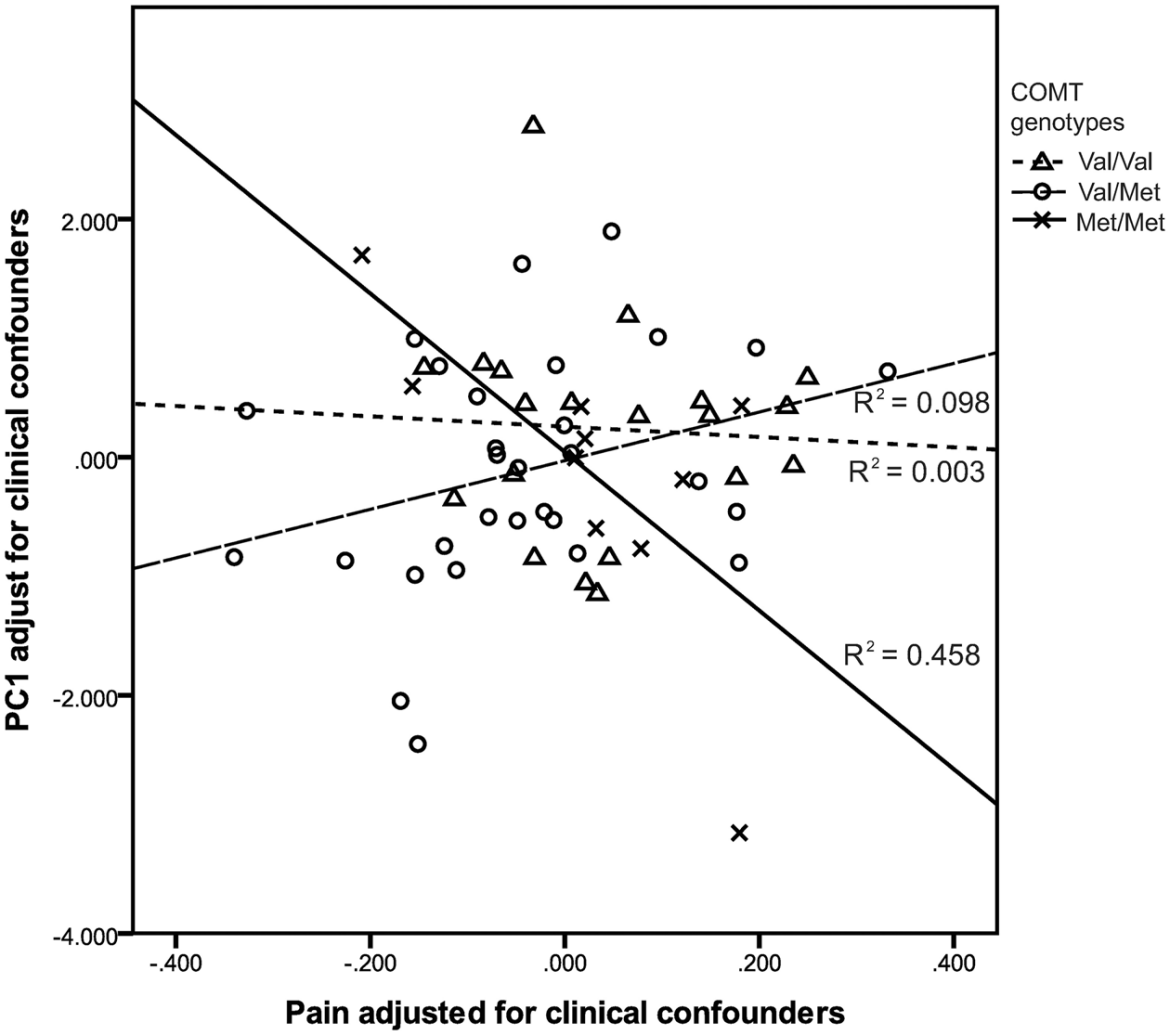
**Greater neonatal pain-related stress was associated with lower *SLC6A4* promoter methylation in very preterm children with *COMT* Met/Met genotype**.

To test our hypothesis that neonatal pain would interact with *COMT* Val158Met genotype to affect *SLC6A4* methylation (PC1), a GZLM model was built, in the very preterm group only, with neonatal pain (cumulative skin breaking procedures from birth to term equivalent age) and *COMT* Val158Met genotypes (Val/Val, Val/Met, Met/Met), and clinical confounders related to prematurity (GA, number of days on mechanical ventilation, SNAP-II day 1, number of surgeries, presence of culture proven infection, cumulative dose of morphine) as covariates. All variables and interactions were entered simultaneously in the model. GZLM results are summarized in Table 6. There was a significant interaction between *COMT* Val158Met genotype and neonatal pain after adjusting for clinical confounders. Greater number of skin breaking procedures (independent of these covariates) was significantly associated with lower overall *SLC6A4* methylation (PC1 of PCA) in children with *COMT* Met/Met genotype (*r*^2^ = 0.46, *p* = 0.001). This interaction is illustrated in Figure 1. Concurrent parental depressive symptoms (BDI-II) and trait anxiety (STAI), hair cortisol level, and *5HTTLPR* variants were included in a further model and none of them yielded significant results (*p* = 0.330, 0.062, 0.142, and 0.388 respectively).

**Table 6:** **Generalized linear modeling analysis of *SLC6A4* methylation predicted by *COMT* genotype and neonatal pain-related stress**.

**Parameter**	**B^a^**	**Std. error**	**95% confidence interval**		***p*-Value**
			**Lower**	**Upper**	
COMT Val/Val × Skin-breaks	−0.401	1.3908	−3.127	2.325	0.773
COMT Val/Met × Skin-breaks	1.550	0.9558	−0.324	3.423	0.105
COMT Met/Met × Skin-breaks	−5.634	1.6642	−8.896	−2.373	**0.001**
COMT Val/Val	−10.012	4.0286	−17.908	−2.116	**0.013**
COMT Val/Met	−14.469	3.4845	−21.298	−7.639	**0.000**
Neonatal infection	0.542	0.3137	−0.073	1.157	0.084
Cumulative morphine dose	−0.237	0.7364	−1.681	1.206	0.747
Days of ventilation	0.006	0.0153	−0.024	0.036	0.710
SNAP-II day 1	0.016	0.0132	−0.010	0.042	0.216
Number of surgery	−0.007	0.2234	−0.445	0.431	0.976
Gestational age (GA)	0.150	0.1057	−0.057	0.357	0.155

*GZLM results showing SLC6A4 methylation was predicted by COMT genotype and neonatal pain-related stress*.

*Model: SLC6A4 Methylation = (COMT genotype × skin-breaks) + Infection + COMT genotype + skin-breaks + morphine exposure + days of ventilation + SNAP-II + surgery + GA*.

a *Unstandardized regression coefficient*.

*SNAP-II, score for neonatal acute physiology (severity of illness index)*.

*Skin-breaks (i.e., neonatal pain-related stress) is equal to the number of skin-breaking procedures from birth to term equivalent age or hospital discharge; cumulative morphine dose is equal to daily morphine exposure adjusted for daily weight; postnatal infection is counted as the presence of a culture proven infection*.

## Discussion

This is the first study, to our knowledge, to demonstrate that children born very preterm have higher *SLC6A4* promoter methylation compared to full-term children. Previous studies have shown effects of early life adversity on overall higher methylation in *SLC6A4* promoter in adults who were adopted in infancy (Beach et al., 2010) and in patients with major depression (Kang et al., 2013) and bipolar disorder (Sugawara et al., 2011; Zhao et al., 2013). Moreover, our findings suggest that the stress of repeated invasive procedures that very preterm infants are exposed to while in the NICU during the first weeks to months of life may be one of the contributing factors affecting *SLC6A4* methylation in the very preterm children at entry to school age. However, *SLC6A4* methylation was reported to be related to various other concurrent environmental stressors (Zhao et al., 2013). Therefore, in the present study we examined and adjusted for potential effects of concurrent stressors while investigating effects of early pain-related stress on *SLC6A4* methylation at age 7 years.

We found that in very preterm (but not full-term) children, higher methylation of the *SLC6A4* promoter region at age 7 years was associated with overall child behavior problems, after adjusting for concurrent parent stressors, child cumulative cortisol level, and the child *5HTTLPR* genotype. The CBCL Total Problems T score was used as an index of overall emotional and behavioral problems of the child (Achenbach and Rescorla, 2000), and is associated with both concurrent and life time exposure to environmental stress (Compas et al., 1989). The children's higher order-factors of internalizing and externalizing problems were not independently found to be related to *SLC6A4* methylation after adjusting for multiple comparisons. Again, the fact that the adjusted CBCL Total Problems scores were related to the overall *SLC6A4* methylation only in very preterm children suggests a unique pathway to altered *SLC6A4* methylation in these children. We then identified that among the neonatal factors to which only the preterm infants are exposed, greater procedural pain exposure was associated with higher child behavior problems after controlling for concurrent stressors and *5HTTLPR* genotype. As has been established in the literature, lower gestational age (Loe et al., 2011) and maternal depression (Beck, 1999) both are associated with behavior problems in children. Importantly, early adversity of repeated pain-related stress early in life predicted child behavior problems above and beyond these other risk factors. In the same cohort of very preterm children, we have previously reported that greater neonatal procedural pain-related stress exposure was associated with higher internalizing behaviors at 18 months and at school age (Vinall et al., 2013b; Ranger et al., 2014), after accounting for similar clinical factors related to prematurity. In the present study we focused on exploring the relationship between neonatal pain (adjusted for clinical confounders) and *SLC6A4* methylation within the very preterm group in an attempt to further advance our understanding of possible mechanisms that could explain the relationship between prematurity and child behavioral problems.

In children born very preterm we found that greater neonatal pain (adjusted for clinical confounders) was significantly associated with lower methylation, however this was only seen in those children with the *COMT* 158 Met/Met genotype. Previous studies in healthy adults have reported that homozygosity for the *COMT* 158 Met-allele was associated with greater pain sensitivity compared to individuals with the Met/Val and Val/Val genotypes (Zubieta et al., 2003). Therefore, it is possible that the *COMT* 158 Met/Met genotype may be more susceptible (or vulnerable) to neonatal pain. The lower SLC6A4 methylation in children with the Met/Met genotype may reflect a stress-sensitive genetic variation by which the already heightened response to early life stress/pain of these children was biologically reduced. Accordingly, it appears that *COMT* 158, a key dopamine-regulatory allele, may contribute to vulnerability or resilience to early life procedural stress during a period of physiological immaturity. However, in the current study, it is not possible to determine the functional effects of *SLC6A4* methylation and whether increases or decreases in *SLC6A4* methylation have positive or negative implications during childhood. Given that the *COMT* Val158Met genotype (Met/Met) differentially interacted with early exposure to neonatal pain to affect subsequent methylation of *SLC6A4*, we speculate that lower *SLC6A4* methylation in very preterm children with the *COMT* 158 Met/Met genotype suggests a possible resiliency or protective mechanism for children who are exposed to early stress (Armbruster et al., 2012). One possible explanation is the increased cognitive stability linked to *COMT* 158 Met-allele as reported previously in infants (Markant et al., 2014), children (Diamond et al., 2004), and adults (Barnett et al., 2007). According to the tonic/phasic dopamine theory, Met allele may enhance tonic dopamine while Val enhances phasic dopamine. Phasic dopamine may be important updating working memory traces, via D_2_ receptors, while tonic dopamine may enhance stability of traces, via D_1_ receptors (Cohen et al., 2002). The sustained D_1_ activation (tonic dopamine) helps prevent “uncontrolled, spontaneous activation of task-irrelevant representation,” (Durstewitz et al., 2000; Durstewitz and Seamans, 2002) and may function as a buffer against stressful early environment (Bilder et al., 2004).

The present study investigated the methylation status of the *SLC6A4* promoter from saliva samples, since serotonergic neurons cannot be examined (Durstewitz and Seamans, 2002). Previous studies have found relationships between blood DNA methylation and mental disorders such as depression (Philibert et al., 2007; Devlin et al., 2010; Ghadirivasfi et al., 2011; Nohesara et al., 2011). Genome-wide methylation study revealed the DNA methylation levels between blood and postmortem brain tissues to be highly correlated (*r* = 0.90) (Horvath et al., 2012). Another genome-wide DNA methylation study has shown that DNA from white blood cells and saliva have 97% of genes with similar methylation patterns, and saliva samples consistently had lower methylation levels than blood samples (Thompson et al., 2013). Also, methylation of *MB-COMT* from DNA in postmortem brain tissues and saliva were found to be closely related (Nohesara et al., 2011). Therefore, our use of saliva methylation status is justified. In this study, the overall methylation differences between full-term and very preterm children were small but the relative percentage differences between the groups were in the same range as found by others in different populations (e.g., Devlin et al., 2010; Kang et al., 2013).

Our findings suggest that the highly stressful environment of repeated exposure to procedural pain-related stress that very preterm infants undergo during their stay in the NICU appears to be one of the factors contributing to the higher *SLC6A4* methylation and greater behavioral problems at school-age in children born very preterm, compared to full-term. Through their prolonged hospitalization during a vulnerable period of physiological immaturity, very preterm neonates are exposed to multiple factors that appear to alter their neurodevelopmental trajectory, and teasing out specific pain-related effects is challenging. There are other factors such as prenatal parent mental health that may impact prematurity itself, as well as methylation status of offspring. Prenatal and postnatal clinical factors, treatments, and genetic variation might interact or may lead to similar end points, which makes them difficult to isolate (Grunau et al., 2013; Ranger et al., 2014). Future studies are needed to evaluate whether interventions to manage neonatal pain may ameliorate long-term epigenetic changes in very preterm infants.

## Conclusion

This study suggests that early repeated procedural pain-related stress exposure is associated with altered methylation of the *SLC6A4* promoter in children born very preterm, but only among individuals with the *COMT* 158 Met/Met genotype. We found a complex relationship between epigenetic modifications, *COMT* Val158Met genotype and early exposure to highly stressful environmental events that induce pain in children born during a critically sensitive developmental period. These findings may indicate that preterm infants with the *COMT* Val158Met genotype experience pain differently and as a result the long term biological impact of early pain-stress differ and could have implications for subsequent serotonin signaling. These findings begin to address mechanisms underlying pathways leading to behavioral problems or resiliency in this vulnerable population.

### Conflict of interest statement

The authors are responsible for the reported research. We have participated in the concept and design, acquisition of the data, analysis and interpretation of data, as well as drafting and/or revising the manuscript. None of us has any affiliation, financial agreement, or other involvement that would place us in conflict of interest with this manuscript. The authors declare that the research was conducted in the absence of any commercial or financial relationships that could be construed as a potential conflict of interest.

## Abbreviations

NICU: neonatal intensive care unit
HPA: hypothalamic-pituitary-adrenal axis
5HTTLPR: serotonin-transporter-linked polymorphic region
CpG: cystosine-guanosine dinucleotide pairs
DA: dopamine
COMT: catechol-*O*-transferase
PFC: prefrontal cortex
PCA: principal component analysis
GZLM: generalized linear modeling.
